# *CORR* Insights^®^: Perioperative Risk Adjustment for Total Shoulder Arthroplasty: Are Simple Clinically Driven Models Sufficient?

**DOI:** 10.1007/s11999-016-5199-z

**Published:** 2016-12-19

**Authors:** Chris Peach

**Affiliations:** Department of Shoulder and Elbow Surgery, University Hospital of South Manchester, Southmoor Road, Manchester, M23 9LT UK

## Where Are We Now?

With increasing pressure on healthcare budgets around the globe, it is vital for healthcare providers to demonstrate that their procedures deliver value [8]. If we want to improve the value of healthcare, we will need to institute substantial cost-saving measures [3]. Adverse events in hospitals are estimated to affect one in 10 patients [2], and the economic impact of events like infections, adverse-drug events, and surgical complications is substantial. If we could reduce their frequency, cost savings would likely follow.

There are potential risk factors for complications following total shoulder arthroplasty [1], a procedure that has more than tripled in incidence in the last 10 years [6]. Reducing adverse events and readmissions after surgery are two key areas that warrant close attention when improving surgical services [4, 5].

The current study by Bernstein and colleagues compared two models that predict unplanned readmission rates and adverse events after total shoulder arthroplasty. Traditionally, clinicians identify potential risk factors by highlighting the phenotypic traits of their patients and subjecting the data to regression analyses. The disadvantage to this is that other factors deemed unimportant might be overlooked. By preoperatively identifying patients with characteristics that might lead to a higher risk of adverse events or readmissions, these models can potentially modify the risk factors ahead of treatment.

## Where Do We Need To Go?

According to the current study, statistically derived risk-stratification models perform better than those derived from clinical suspicion alone. However, it is still unclear how accurate, comprehensive, or appropriate the variables collected within large databases can be. When working with large databases, we have to balance the inclusion of too many variables (risking in inclusion of inaccurate data), with the omission of data that might identify crucial clinical characteristics.

One area of concern is knowing what to do with the risk factors identified in the preoperative period. Some might be modifiable, such as those in studies that demonstrated the benefits of quitting smoking [8]. However, many of the variables identified in the current study may not be modifiable. Does this mean that we prevent elderly males from undergoing total shoulder arthroplasty due to their risk for complications and readmissions? Do we introduce an additional cost proportional to their identified high-risk status? With increased operating time identified as a risk factor for adverse events in this study, should we be assessing surgeons’ speed of operating? (I suspect this statistic relates to intraoperative complications slowing operative time, rather than the sluggishness of the clinician.)

Data from large databases is only an asset if it is relevant and usable. To enhance the data we extract from such databases, it will be essential to gain a consensus on content and variables that need collecting. We also need to know how to effectively process data to provide knowledge for clinicians and benefits for our patients.

## How Do We Get There?

A previous study on national arthroplasty registers highlighted the importance of a collaborative approach to using databases [7]. However, this study highlighted the inconsistences of datasets from various contributing sources. We need to aspire towards international standardization of the most appropriate variables to include in large databases. There will need to be thorough validation of these items, how they relate to patient outcomes, and their relevance in the population of interest. The content of databases should be developed through collaboration between national societies, organizations and registry groups which in turn should improve standards of data collections and data quality.

Instead of needing copious randomized trials to explore the concept of risk stratification and “evidence-based practice,” it will be “practice-based evidence”—using robust, prospectively maintained databases—that will prevail and be the more successful strategy. Prospective studies could identify strategies to modify the risk factors associated with total shoulder arthroplasty, as well as determine whether identifying risk factors does indeed modify the outcome for this patient population.
